# Outcomes of a modified technique of partial parotidectomy and novel parotid tumour position classification from a single surgeon prospective database

**DOI:** 10.1111/ans.19261

**Published:** 2024-10-09

**Authors:** Jonathan W. Serpell, Zelia K. Chiu, Edward Forrest, James C. Lee

**Affiliations:** ^1^ Department of Surgery The Alfred Hospital Melbourne Victoria Australia; ^2^ Department of Surgery Central Clinical School, Monash University Melbourne Victoria Australia

**Keywords:** facial nerve palsy, nerve monitoring, parotid tumour site classification, parotid tumours, parotidectomy

## Abstract

**Background:**

Conservative parotidectomy for benign tumours reduces facial nerve palsy, without increasing local recurrence. We report a modified technique of partial parotidectomy and using a novel description of tumour position, explore relationships between tumour position and histological margins, facial nerve palsy and local recurrence.

**Methods:**

A prospectively collected single surgeon parotidectomy database was analysed, including tumour location (superficial/deep lobe; central/peripheral) and outcomes. A partial parotidectomy identified the facial nerve and the proximal portion of its branches with a macroscopically clear resection margin. Mean follow up was 5.9 years for pleomorphic adenomas.

**Results:**

Three hundred and three patients underwent parotidectomy; 257 (84.8%) were superficial and 46 (15.2%) deep lobe. Tumour position was recorded in 291: 236 (81.1%) were peripheral tumours and 55 (18.9%) central. Histological margin involvement was similar in central and peripheral tumours, both overall and for superficial and deep lobe tumours, but was commoner in central deep lobe tumours, (*P* = 0.003). Temporary partial facial nerve palsy occurred in 21 (6.9%), with one permanent partial nerve palsy (0.3%). Deep lobe tumours and total parotidectomy were associated with facial nerve palsy (*P* = 0.01). Facial nerve monitoring reduced the risk of palsy (*P* < 0.01). Local recurrence of pleomorphic adenomas was uncommon, occurring in 3 (2.0%) of 151 patients.

**Conclusion:**

This series confirms the safety and adequacy of more conservative partial parotidectomy for benign tumours, highlighting most tumours are peripheral, but not more prone to histological margin involvement or local recurrence, and with routine intraoperative facial nerve monitoring, is achieved with low facial nerve palsy rates.

## Graphical abstract

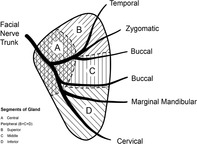

## Introduction

Previously, complete superficial parotidectomy has been considered the gold standard of parotid surgery, to ensure wide margins are achieved around a parotid lesion, thereby reducing the risk of local recurrence of a parotid tumour. However, this procedure had a relatively high associated risk of facial palsy, variably reported between 8% and 32%, but with some series even higher at up to 68%. Risk factors for complications of parotid surgery, such as facial nerve palsy, are difficult to establish due to the uncommon nature of parotid tumours and the resultant small study sizes but include extent of surgery – partial or complete superficial or total parotidectomy. Consequently, since 2003 there has been increasing adoption of more conservative parotid resections to reduce morbidity, especially facial nerve palsy, without increasing local recurrence.^1^, ^2^, ^3^, ^4^, ^5^, ^6^, ^7^, ^8^, ^9^, ^10^, ^11^, ^12^, ^13^, ^14^, ^15^

A variety of forms of more conservative parotidectomy are described. These include limited parotidectomy (LP) involving a focused tumour dissection, identifying the facial nerve trunk, but only the branches related to the tumour, and removing a margin of normal parotid tissue around the tumour.^16^ Extracapsular dissection involves removal of only a rim, usually of 2–3 mm, of healthy parotid tissue circumferentially, without formal facial nerve identification.^8^, ^17^ Focused tumour dissection does not identify the facial nerve trunk but does dissect branches related to the tumour.^18^ These more conservative parotidectomies report lower facial nerve palsy rates of 1.1%–9% when removing small benign pathologies but with a low risk of tumour recurrence.^4^, ^19^

The aims of this study were to report our modified technique and results of partial parotidectomy, which is similar to the LP technique, but differs in that the trunk and all branches of the facial nerve are identified and dissected to a limited extent, and using a novel description of tumour position identified intraoperatively (central versus peripheral), to explore relationships between tumour position and histological margins, local recurrence, and facial nerve palsy.

## Materials and methods

### Data collection, inclusions and exclusions

A prospective single surgeon parotidectomy database of patients entered between 1993 and 2022, was interrogated to identify adult patients (age > 18 years). Only patients to the end of 2022 were included to allow a minimum of 12 months follow up. Only superficial and deep lobe tumours were included; parapharyngeal tumours were excluded.

The database collects baseline demographics, preoperative assessment, operative details, pathology, postoperative complications, follow up and outcomes, including local recurrence.

The primary outcome measures were tumour position – central versus peripheral, and histological margin involvement, and secondary outcomes were facial nerve palsy and local recurrence.

### Operation

We removed superficial lobe tumours following identification of the facial nerve trunk and the proximal portion of all its five main branches, but without a complete dissection of the full extent of the branches, and then excised the tumour with a macroscopic margin of normal parotid tissue. The facial nerve and its branches were meticulously dissected with fine mosquito forceps, lifting small amounts of parotid tissue anterograde, which were then ligated with fine absorbable ties, and divided sharply. Blunt dissection was not utilized. Haemostasis was also obtained with bipolar diathermy, but energy vessel sealing devices were not used. The parotid duct was not usually identified or ligated. Tumours abutting the facial nerve, or its branches were shaved off intact, without breaching the capsule. Hence, most had a wide local excision or partial superficial lobe parotidectomy. Formal complete superficial parotidectomy was rarely performed. No tumours were enucleated. Deep lobe tumours were removed by resection of part of the superficial lobe to expose the relevant facial nerve branches and then the deep lobe tumour was excised (near total parotidectomy). Suction drainage was routinely used for 24 h.

### Description of tumour position

**Fig. 1:**
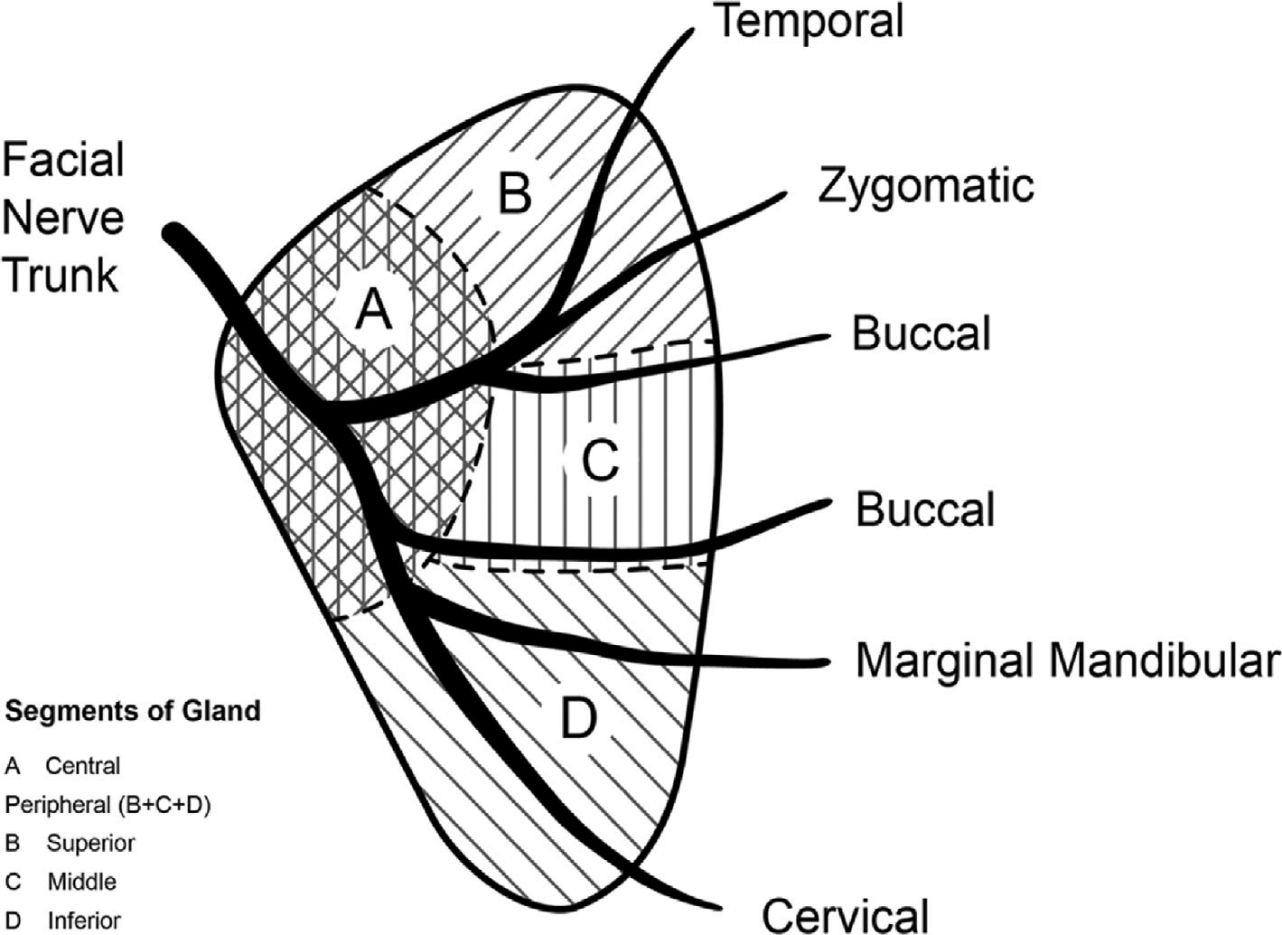
Schematic of parotid gland, facial nerve and central and peripheral classification.

We defined tumour position intraoperatively: a central tumour was located at the trunk or bifurcation of the facial nerve (temporo‐zygomatic and cervico‐facial divisions); a peripheral tumour was located at or distal to the facial nerve divisions (therefore related to the 5+ terminal branches). Peripheral tumours were sub‐classified into superior (related to temporal and/or zygomatic branches), middle (related to buccal branches) and inferior (related to marginal‐mandibular and/or cervical branches). This tumour description is shown in the schematic diagram, Figure 1. We applied the tumour position description to both superficial and deep lobe tumours. Throughout, results are presented classified by tumour position.

### Follow up

We aimed to follow patients with pleomorphic adenomas clinically 12 monthly for at least 5 years, and preferably 10 years, if tumour was abutting facial nerve branches or with close margins. We analysed follow up for pleomorphic adenomas which are at risk of local recurrence to assess the outcome of our more conservative parotidectomy. We did not follow up other benign tumours such as Warthin's or cysts which do not recur.

### Statistical analysis

All data are presented as mean and standard deviation (SD) or median and interquartile range (IQR). Chi squared was used to compare differences between discrete variables. Univariate logistic regression analysis was conducted for factors predicting facial nerve palsy. A two‐sided p value of less than 0.05 was considered statistically significant. All statistical analysis was performed using Stata/IC version 14.2 (StataCorp 2016, College Station, TX, USA).

Ethics was approved by the Alfred Human Research Ethics Committee.

## Results

There were 303 patients, 160 men (52.8%) and 143 women (47.2%), of mean (SD) age 57.8 (16.5) years. 257 (84.8%) underwent partial superficial lobe parotidectomy, and 46 (15.2%) near total parotidectomy for deep lobe tumours (Table 1).

**Table 1:** Patient demographics

Lobe	Superficial	Deep	Total
Age (years)			
Range	17–90	20–87	17–90
Average	58	59	57.8
Median	60	62	60
Sex			
Female	123	20	143
Male	134	26	160
Total	257	46	303

Tumour position was available for analysis in 291 (96%) cases. 236 (81.1%) were peripheral tumours and 55 (18.9%) central. Of the peripheral tumours, 41(17.4%) were superior, 50 (21.2%) middle and 145 (61.4%) inferior. The distribution was similar for superficial and deep lobe tumours (Table 2).

**Table 2:** Tumour position

Lobe	Superficial (%)	Deep (%)	Total (%)
Central	46 (18.6)	9 (20.1)	55 (18.9)
Peripheral	201 (81.4)	35 (79.6)	236 (81.1)
Superior	36 (17.9)	5 (14.2)	41 (17.4)
Middle	41 (20.4)	9 (25.7)	50 (21.2)
Inferior	124 (61.7)	21 (60.0)	145 (61.4)
Position unknown	10 (3.9)	2 (4.3)	12 (4.0)
Site recorded	247 (96.1)	44 (95.7)	291 (96)
Total	257	46	303

Two hundred and sixty‐five tumours (87.5%) were benign and 38 (12.5%) malignant. The commonest were pleomorphic adenomas (*n* = 151, 49.8%) and Warthin's tumour (*n* = 67, 22.1%). The mean (SD) maximal tumour diameter was 23.0 mm (12.4 mm) (Table 3).

**Table 3:** Histopathology parotid tumours and tumour size

Benign	Superficial (222)	Deep (43)	Total (265) (87.5%)
Pleomorphic Adenoma	121	30	151 (49.8%)
Warthin's	60	7	67 (22.1%)
Cyst	8	1	9 (3.0%)
Other	33	5	38 (12.5%)
Malignant	Superficial (35)	Deep (3)	Total (38) (12.5%)
Lymphoma	8	0	8 (2.6%)
2° SCC	12	0	12 (4.0%)
2° Melanoma	7	0	7 (2.3%)
Acinic cell tumour	3	0	3 (0.99%)
Muco‐epitheloid	3	1	4 (1.3%)
Other	2	2	4 (1.3%)
Total	257	46	303
Tumour size (mm)			
Lobe	Superficial	Deep	All
Mean	23.0	24.0	23.0
Median	20.0	24.0	22.0
Range	2.8–75.0	6.0–50.0	2.8–75.0

Histological margins are detailed in the 291 cases where tumour position was available. Of these, 34 (11.7%) were reported with histologically involved margins; 9.3% (23/247) of superficial lobe lesions and 25% (11/44) of deep lobe lesions (*P* = 0.003) (Table 4). All cases of margin involvement histologically had intact capsules macroscopically and were shaved off facial nerve branches, or were at the periphery of the gland as described above.

**Table 4:** Histological margins versus tumour position

Lobe	Superficial (257)		Deep (46)		Total (303)	
	*N*	Involved margin (%)	*N*	Involved margin (%)	*N*	Involved margin (%)
Central	46	2 (4.3)	9	4 (44.4)	55	6 (10.9)
Peripheral	201	21 (10.4)	35	7 (20)	236	28 (11.9)
Superior	36	3 (8.3)	5	1 (20)	41	4 (9.8)
Middle	41	4 (9.8)	9	1 (11.1)	50	5 (10)
Inferior	124	14 (11.3)	21	5 (23.8)	145	19 (13.1)
Position Unknown	10	0 (0)	2	0 (0)	12	0 (0)
Total Known	247	23 (9.3)	44	11 (25.0)	291	34 (11.7)

The tumour site (central versus peripheral) was not associated with increased margin involvement. Overall margin involvement was similar in central (6/55, 10.9%) and peripheral tumours (28/236, 11.9%) (*P* = 0.84) for the total series, and for superficial (4.3% versus 10.4%, *P* = 0.20) and deep lobe tumours (44.4% versus 20%, *P* = 0.30). Margin involvement was not significantly different in the peripheral subdivisions (*P* = 0.8). Margins were involved in significantly more deep lobe central tumours (44.4%) compared to superficial lobe central tumours (4.3%), (*P* = 0.003). Margin involvement for peripheral tumours was not significantly different in deep lobe compared to superficial lobe tumours (20% versus 10.4%, *P* = 0.11) (Table 4).

Of those with positive margins, 27 (79.4%) were benign; 19 pleomorphic adenomas and 8 Warthin's tumours. Of the 27 benign lesions, 63% were in the superficial lobe and 37% in the deep lobe (Table 5).

**Table 5:** Pathology of tumours with positive margins

Lobe	Superficial	Deep	Total
Pleomorphic adenoma	11	8	19
Warthin's	7	1	8
Other	5	2	7
Total	23	11	34

Twenty‐one (6.9%) patients had a temporary partial facial nerve palsy. The rate was similar for superficial and deep lobe tumours (6.2% versus 10.9%, *P* = 0.21) (Table 6). The commonest was marginal mandibular, comprising 51.2% of all partial facial nerve palsies. 1 (0.3%) patient had a permanent partial palsy (marginal mandibular branch only). There were no total facial nerve palsies. Intra‐operative nerve monitoring (IONM) was routinely utilized since 2007, in 238 (78.5%) cases.

**Table 6:** Facial nerve palsy

Lobe	Superficial (257) (%)	Deep (46) (%)	Total (303) (%)
Temporary facial nerve palsy	16 (6.2)	5 (10.9)	21 (6.9)
Central	4 (1.6)	1 (2.2)	5 (1.7)
Peripheral	12 (4.7)	4 (8.7)	16 (5.3)
Superior	0 (0)	3 (6.5)	3 (1)
Middle	4 (1.6)	0 (0)	4 (1.3)
Inferior	7 (2.7)	1 (2.2)	8 (2.6)
Permanent facial nerve palsy	0 (0)	1 (2.2)	1 (0.3)
Central	0 (0)	0 (0)	0 (0)
Peripheral	0 (0)	1 (2.2)	1 (0.3)
Superior	0 (0)	1 (2.2)	1 (0.3)
Middle	0 (0)	0 (0)	0 (0)
Inferior	0 (0)	0 (0)	0 (0)

A univariate regression analysis identified deep lobe tumours (*P* = 0.04) and near total parotidectomy (*P* < 0.04) were associated with facial nerve palsy; and IONM significantly decreased the risk of facial nerve palsy from 15.4% (10/65) to 4.3% (10/235) (*P* < 0.01).

Wound infection occurred in 11 (3.6%) and salivary fistula in 23 (7.6%) patients but resolved in all by 6 weeks. One (0.3%) patient had a sialocele requiring repeat aspiration which resolved after 2 months.

After excluding patients with benign lesions which do not recur (including Warthin's, cysts, lipomas, sialadenitis, and myoepitheliomas), and malignancies, we analysed the 151 pleomorphic adenomas at risk of local recurrence. Of these, follow up data where greater than 12 months was available in 127(84.1%) of the 151 cases of pleomorphic adenoma – for this group the mean follow up was 5.9 years (median 5 years, range 1–19 years). Local recurrence was uncommon – 3 patients (2.0%) had a recurrence of pleomorphic adenoma – one of these had a positive histological margin: none were deep lobe tumours, 2 were central and 1 peripheral (Table 7).

**Table 7:** Local recurrence pleomorphic adenomas (*N* = 151)

Lobe	Superficial (121) (%)	Deep (30) (%)	Total (151) (%)
Recurrence	3 (2.5)	0 (0)	3 (2.0)
Central	2 (1.3)	0 (0)	2 (1.3)
Peripheral	1 (0.8)	0 (0)	1 (0.7)
Superior	1 (0.8)	0 (0)	1 (0.7)
Middle	0 (0)	0 (0)	0 (0)
Inferior	0 (0)	0 (0)	0 (0)

## Discussion

We have documented a novel intraoperative description of the anatomical position of parotid tumours, highlighting peripheral and central tumour locations in superficial and deep lobes, and analysing histological margins, facial nerve palsy and local recurrence in relation to tumour position, in contrast to other classifications which describe surgical extent of parotidectomy.^20^

More conservative parotid resections aim for a complete tumour resection without formal superficial parotidectomy, and a low facial nerve palsy rate. In this series we report our modified technique of identifying the facial nerve trunk first and the proximal portion of all five main branches, followed by resection of parotid around the tumour to achieve a wide local excision or partial parotidectomy, but without completely exposing the full extent of the branches. Our technique differs from limited parotidectomy, where only the branches of the facial nerve related to the tumour are dissected.^16^

With benign parotid tumours, particularly pleomorphic adenomas, the extent of parotidectomy is that required to achieve an adequate margin around the tumour, aiming to achieve a low local recurrence rate. However, as this series shows, 81.1% of tumours are peripherally situated. Peripheral tumours have a narrower margin of available surrounding tissue for resection (as they are at the margin of the parotid gland) compared to central tumours. Therefore, recognizing most tumours are peripheral enables an awareness of the proximity of the tumour capsule to the margin of the parotid gland, and therefore assists planning of surgical resection, achieving a clear margin without breaching the tumour capsule. Despite the commonness of peripheral location, this series shows histological margin involvement was no greater in the peripheral compared to central tumour location, for the total series and when analysing superficial lobe and deep lobe tumours individually. The rate of histological margin involvement was similar for peripheral superficial compared to peripheral deep lobe tumours, but margin involvement was commoner in central deep compared to central superficial tumours.

Focal capsular exposure requiring controlled dissection or tumour shave occurs in almost all parotidectomies, usually of the facial nerve or its branches.^16^, ^21^ Hence, even when a cuff of normal tissue around a tumour and macroscopic tumour clearance is achieved, there will often be a point where the tumour is close or at the margin of resection on a nerve or at the periphery of the parotid gland. However, this series reports a relatively low histologically involved margin rate of 11.7%, compared to rates of 25%–73% in the literature.^8^, ^22^ Despite this, low rates of local recurrence can be achieved, such as 2.0% for pleomorphic adenomas in this series, with these more conservative parotidectomies, in contrast to the historical procedure of intracapsular enucleation which led to local recurrence rates of up to 45%.^21^

Therefore, this series adds to the current literature that complete local excision achieving clear macroscopic margins at operation, using our modified technique of partial parotidectomy, adequately treats benign parotid tumours, without the need for complete superficial parotidectomy, and results in a low local recurrence rate of 2.0%. This rate is concordant with current reports of 2%–5%.^21^

Our tumour position description differs from that developed by the Spanish group in 2010, and further adopted by the European Salivary Gland Society (ESGS) in 2016.^20^, ^23^ Our system recognizes three peripheral tumour levels related to the upper and lower divisions of the facial nerve trunk, but also recognizing a middle component related to buccal branches. In contrast, the ESGS classification aims to describe extent of parotid resection but only subclassifies the peripheral portion into lateral superior and lateral inferior. The key difference between the two classifications is that our system describes tumour position in detail, whereas the ESGS system describes surgical extent of parotidectomy. This series has confirmed the known distribution of parotid tumours, for example that the commonest site for tumours is superficial lobe, peripheral and inferior.

This series reports low rates of facial nerve palsy, with 6.9% temporary and 0.3% permanent partial facial nerve palsy, compared to reported rates of 1.1%–9% and 4% respectively in the literature.^4^, ^12^, ^19^ This low facial nerve palsy rate adds to the literature that more conservative, partial parotidectomies reduce palsy rates due to reduced facial nerve exposure compared to a more extensive complete superficial parotidectomy.^16^ IONM was associated with decreased facial nerve palsy: we recommend its routine use, as although identification of the facial nerve trunk relies on surgical technique, we believe IONM assists preserving the branches of the facial nerve.^2^, ^24^ The reduction in palsy rate with IONM in this series from 15.4% to 4.3% is consistent with a meta‐analysis by Sood showing IONM halved facial nerve palsy rates.^25^

Deep lobe tumours were associated with poorer patient outcomes. Central deep lobe tumours had a greater association with margin involvement compared to central superficial lobe tumours. Further, deep lobe tumours and near total parotidectomy were associated with temporary partial facial nerve palsies, a finding reported by others.

This series has shown relatively low rates of wound infection (3.6%), salivary fistula (7.6%) and sialocele (0.3%), similar to reports in the literature.^16^, ^18^

This study has some limitations. First, it is retrospective. However, one of its strengths is that the data was extracted from a carefully designed, prospectively collected parotidectomy database, with single surgeon follow up. Second, the size of this series (303 cases) is in the middle range of other reports – 70 – 794 cases^2^, ^19^. Third, the mean follow‐up for pleomorphic adenomas in this series is 5.9 years, but it is well known local recurrence may occur decades later. However, most parotidectomy series have a similar follow up, with only a minority greater than 10 years.^15^, ^18^

## Conclusions

This series confirms the safety and adequacy of more conservative partial parotidectomy for benign tumours, highlighting most tumours are peripherally situated, but not more prone to positive histological margin involvement. Partial parotidectomy with routine intraoperative facial nerve monitoring, is achieved with low facial nerve palsy rates, and low local recurrence.

## Conflict of interest

Jonathan Serpell is an Editorial Board member of ANZJSurgery and a co‐author of this article. To minimize bias, they were excluded from all editorial decision‐making related to the acceptance of this article for publication.

## Author contributions

**Jonathan W. Serpell:** Conceptualization; data curation; formal analysis; methodology; project administration; resources; software; supervision; validation; writing – original draft; writing – review and editing. **Zelia K. Chiu:** Data curation; formal analysis; writing – original draft; writing – review and editing. **Edward Forrest:** Data curation; formal analysis; writing – original draft; writing – review and editing. **James C. Lee:** Formal analysis; supervision; writing – original draft; writing – review and editing.
